# Deep roots and soil structure

**DOI:** 10.1111/pce.12684

**Published:** 2016-02-05

**Authors:** W. Gao, L. Hodgkinson, K. Jin, C.W. Watts, R.W. Ashton, J. Shen, T. Ren, I.C. Dodd, A. Binley, A.L. Phillips, P. Hedden, M. J. Hawkesford, W.R. Whalley

**Affiliations:** ^1^ China Agricultural UniversityBeijing100193China; ^2^ Lancaster Environment CentreLancaster UniversityLancasterLA1 4YQUK; ^3^ Huazhong Agricultural UniversityHongshan DistrictWuhan430070China; ^4^ Rothamsted ResearchWest CommonHarpendenSt. AlbansAL5 2JQUK

**Keywords:** penetrometer resistance

## Abstract

In this opinion article we examine the relationship between penetrometer resistance and soil depth in the field. Assuming that root growth is inhibited at penetrometer resistances > 2.5 MPa, we conclude that in most circumstances the increases in penetrometer resistance with depth are sufficiently great to confine most deep roots to elongating in existing structural pores. We suggest that deep rooting is more likely related to the interaction between root architecture and soil structure than it is to the ability of a root to deform strong soil. Although the ability of roots to deform strong soil is an important trait, we propose it is more closely related to root exploration of surface layers than deep rooting.

## Introduction

There is convincing evidence for the benefits of deep rooting, especially in relation to drought resistance (Lopes & Reynolds, 2010; Uga *et al.,* 2013). Modelling has shown that greater root depth allows increased water uptake and higher yields (Lilley & Kirkegaard, 2011). Deep rooting is thought to be improved by combinations of traits that confer steeper growth and an ability to penetrate strong layers (Lynch, 2013). There is a view that natural variability in root depth between species and within the same species (e.g. Canadell *et al.,* 1996), for example, for wheat (*Triticum aestivum*), provides a basis for developing breeding programmes to develop deep‐rooted crops (e.g. Kell, 2011). However, an alternative explanation is the widely reported effect of soil structure on rooting depth (White & Kirkegaard, 2010; Valentine *et al.,* 2012). The primary purpose of this article is to alert plant scientists to the restrictions to deep rooting that are imposed by soil conditions simply by virtue of depth in the profile which has the effect of increasing soil strength because of the combined effects of hydrostatic pressure and internal soil friction (Richards & Greacen, 1986); in doing so we emphasize the role of soil structure. In some respects these are well reported: for example Valentine *et al.* (2012) demonstrated the importance of macro‐pores, while White & Kirkegaard (2010) showed that at depth all roots were found in pre‐existing pores. However, we will argue that in the field the increase in soil strength at depth that occurs irrespective of soil management, must inevitability restrict root growth to existing pore networks. The findings of White & Kirkegaard (2010) showing that deep roots are only found in pores should be considered to be the norm.

## Soil Strength

### Measuring the resistance to penetration in soil

**Figure 1:**
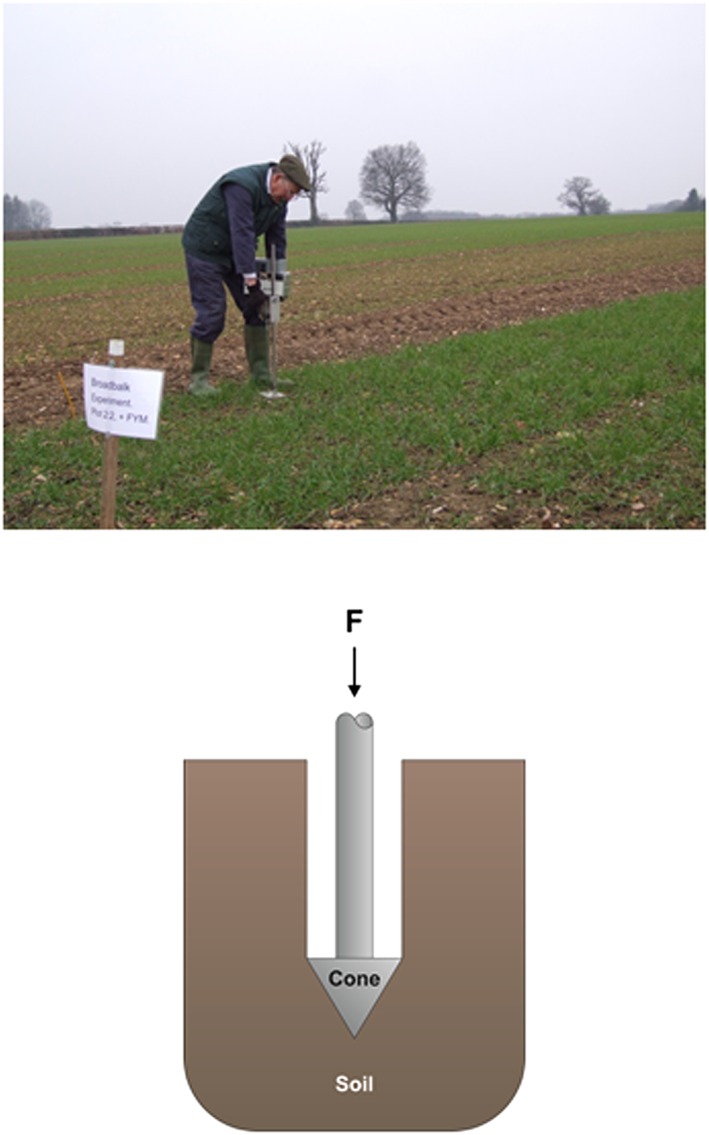
A penetrometer in use in a field to measure the relationship between penetrometer resistance and depth. The insert shows the relieved shaft and a conical cone to deform the soil.

**Figure 2:**
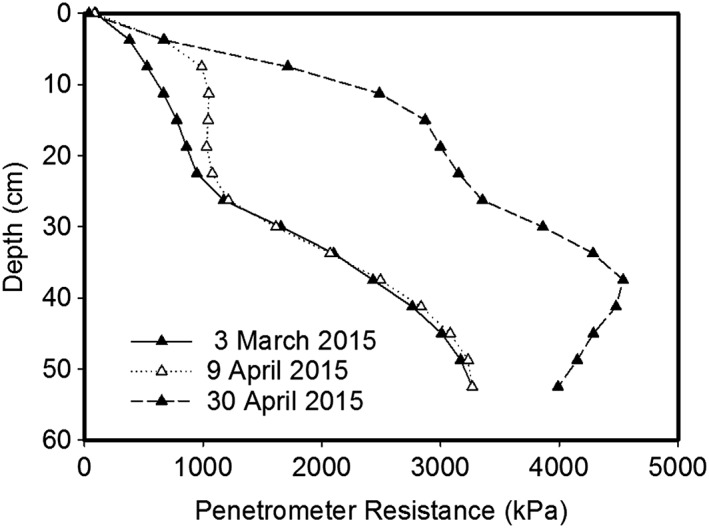
Examples of penetrometer profiles on a silty clay soil at the Rothamsted Experimental farm near Woburn in Bedfordshire. On 3 March, when there had been negligible soil drying, soil penetrometer resistance increased with depth despite little change in soil density or soil moisture with depth. The increases in penetrometer resistance between 3 March and 30 April are because of the effects of soil drying by wheat roots.

An important aspect of understanding the response of roots to strong soil is the ability to conduct laboratory experiments with realistic rooting environments, replicating soil water status, soil strength, oxygen availability and nutrient status experienced in the field. In this article our primary interest is soil strength, and this can be measured with a penetrometer (Fig. 1) both in the lab and the field. In laboratory experiments the elongation rate of roots has been shown to decrease with increasing penetrometer resistance (Bengough & Mullins, 1991). There has been considerable interest in finding relationships between soil properties and penetrometer resistance. It is common practice to measure penetrometer resistance in soil cores, either undisturbed or repacked to a prescribed density, and to develop relationships between penetrometer resistance and various other soil properties (To & Kay, 2005; Whalley *et al.,* 2005; Whalley *et al.,* 2007; Gao *et al.,* 2012; Gao *et al.,* 2016). To an extent this approach has been very successful and the strength in the surface layers of soil can be predicted with empirical models (Gao *et al.,* 2012). However, a problem arises with deeper layers because field data shows that soil at depth is stronger (Fig. 2), which is not taken into account in simple models (Gao *et al.,* 2016). In our view the over‐reliance on relationships between soil penetrometer resistance and other soil conditions (water content, water potential and density) which have been developed with laboratory cores has resulted in the effect of depth on penetrometer resistance being overlooked. However, this effect is well understood by the geotechnical community (e.g. Skempton, 1987) and data such as those shown in Fig. 2, where penetrometer resistance increases with depth, would be considered normal.

### A model for soil penetrometer resistance

**Figure 3:**
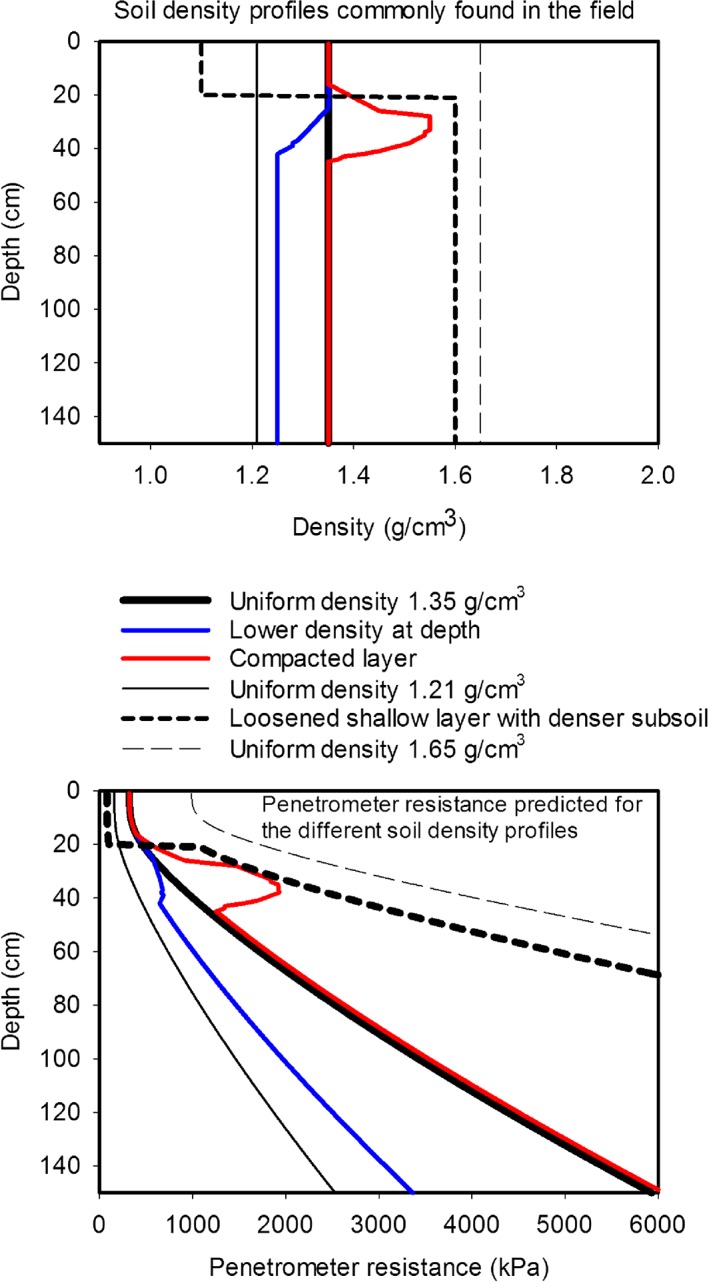
The use of Eqn 1 (Gao *et al.,* 2016) to predict penetrometer resistance profiles for various soil density–depth scenarios in well‐watered soil. These predictions are consistent with data shown in Fig. 2 as well as published data showing increases in penetrometer resistance to values greater than 4 MPa at depths as shallow as 50 cm (e.g. Van Hussteen, 1983; Raper *et al.,* 1999; Tekeste *et al.,* 2008; Chen & Weil, 2009).

Gao *et al.* (2016) have recently proposed the following model to predict soil penetrometer resistance (*Q*),

$$Q=\rho {\left(A^{*}\frac{{\left(F-e\right)}^{2}}{1+e}{\left(\sigma_{s}^{p}-\psi S^{*}\right)}^{f}\right)}^{2}\text{,}$$

in relatively well‐watered field conditions, where *ρ* is the dry bulk density of soil in kN m^−3^, *e* is the void ratio, *σ*_s_ is the net stress (kPa), *ψ* is matric potential (kPa) and where S* = degree of saturation (*S*) if *S* > 0.5, otherwise S* = 0.5 (Gao *et al.,* 2012; Whalley *et al.,* 2012). F, A*, p and *f* are empirical adjustable parameters. They assumed that *σ*_s_ was simply related to the weight of soil above any given depth, and were able to predict penetrometer data obtained in the field. We have compared different soil density profiles which are commonly reported (e.g. Van den Akker & Schjønning, 2004), and show that at depth all soils increase in strength sufficiently (>2500 kPa) to limit root elongation (Fig. 3). The penetrometer resistances in Fig. 3 were predicted using the parameter values reported by Gao *et al.* (2016), and although the predictions may differ for other soil types, the central point that penetrometer resistance increases with depth will be unaffected. We assumed that the soil was well watered and that penetrometer resistance was determined by depth and density, which is the most optimistic scenario with respect to root penetration into strong soil, because drier soils will have a greater penetrometer resistance (Fig. 2). Our predictions show that the most widely reported phenomenon of a compacted layer would indeed affect rooting depth, as is commonly reported (Ball *et al.,* 2015), but even if compaction were completely ameliorated rooting depth would still be restricted. These predictions ignore soil drying, but they do provide realistic descriptions of soil strength profiles of winter wheat in UK conditions. The predictions (Fig. 3) are consistent with the published data (e.g. Van Hussteen, 1983; Raper *et al.,* 1999; Tekeste *et al.,* 2008; Chen & Weil, 2009).

### Deformation of soil by roots

Soil deformation processes that occur around roots are reasonably well understood (Farrell & Greacen, 1966; Greacen *et al.,* 1968; Greacen & Oh, 1972; Richards & Greacen, 1986; Kirby & Bengough, 2002). Advancements in this field have largely depended on using more refined models of soil mechanics, which have informed on the effects of soil to root friction on the axial pressure experienced by the root as it deforms soil (Kirby & Bengough, 2002). The elongation of roots has been shown to be particularly sensitive to axial pressure, while somewhat insensitive to radial pressure (Bengough, 2012). This observation explains why roots are good at exploiting existing pore networks even if they are smaller than the diameter of the root. Interestingly, the maximum growth pressures of roots from very different species are relatively similar (Clark & Barraclough, 1999).

**Figure 4:**
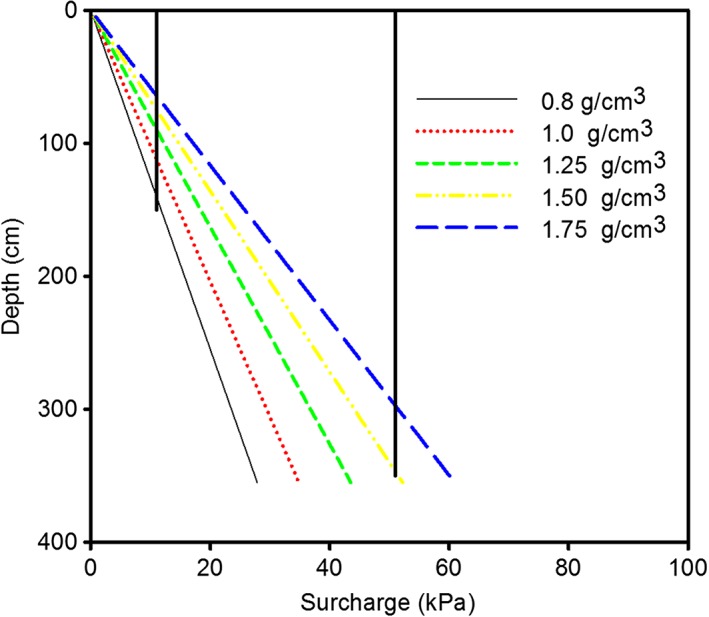
The effect of soil density on surcharge as a function of depth. Also indicated is the pressure applied to sand culture experiments by Coelho Filho *et al.* (2013) and by Materachera *et al.* (1991) to increase the penetrometer resistance of the root growth environment. The effect of this pressure on penetrometer resistance is amplified by the internal friction of soil (Richards & Greacen, 1986).

The effect of soil strength on root and shoot elongation has recently been investigated with sand culture systems (Coelho Filho *et al.,* 2013; Jin *et al.,* 2015a). Here a confining pressure from an axial load was used to increase the mechanical strength of sand to provide a rooting environment that was otherwise well‐watered and well‐aerated. Both Jin *et al.* (2015a) and Coelho Filho *et al.* (2013) applied an axial pressure of 11 kPa to the surface of a sand culture to obtain a high impedance environment which reduced root mass to approximately 30% of its value in the control treatment with no axial pressure. Actually 11 kPa is approximately the axial pressure (or surcharge) that could be expected at a depth of about 80 cm in the field, depending on soil density (Fig. 4). To investigate the response of roots to very strong soil, Materachera *et al.* (1991) used a higher axial pressure (analogous to a greater surcharge) of 51 kPa, corresponding to the effect of surcharge at a depth of approximately 350 cm, although the penetrometer resistance they achieved was approximately 4.2 MPa which is commonly exceeded at much shallower depths (Fig. 2; Van Hussteen, 1983; Tekeste *et al.,* 2008). The elongation recorded by Materachera *et al.* (1991) was no greater than 0.7 mm day^−1^ (for lupin) and in the order of less than 10% of the rate in the absence of impedance (Table 1). These data illustrate how limited root elongation would be at depth in a structureless soil. They also show limited genotypic variation in elongation in uniformly strong soil which is too small to be a useful trait, an observation also made for different rice lines by Clark *et al.* (2002) in much weaker soil.

**Table 1:** Elongation of roots following 10 days of growth in a very strong soil with a penetrometer resistance greater than 4 MPa or a mechanically weak control (from Materachera *et al.,* 1991)

	Root elongation following 10 days of growth (mm)				
Plant species	Strong soil		Weak control		Percentage reduction by stress
Monocotyledons		se		se	
Barley	3.1	0.04	124.6	0.76	97.5
Maize	4.4	0.06	106.7	0.72	95.9
Oats	3.2	0.05	114.2	1.14	97.2
Rice	3.1	0.02	60.2	0.15	94.9
Sorghum	3.4	0.02	63.8	0.15	94.7
Rhodesgrass	2.5	0.05	60.6	0.36	95.9
Ryegrass	3	0.02	68.2	0.28	95.6
Wheat	4.1	0.04	120.7	0.82	96.6
Dicotyledons					
Cotton	4.5	0.02	68	0.2	93.4
Faba bean	6.8	0.03	98.7	0.74	93.1
Lincoln weed	2.7	0.04	59.8	0.25	95.5
Leucaena	5.2	0.05	66.9	0.22	92.2
Lucerne	4.3	0.03	75.9	0.31	94.3
Lupin	7.1	0.06	69.4	0.27	87.8
Medic	4.5	0.03	62.4	0.22	92.8
Oil radish	4.9	0.04	88.3	0.6	94.5
Pea	7	0.04	104.6	0.85	93.3
Pigeonpea	4.6	0.06	72.7	0.2	93.7
Safflower	5.6	0.05	94.5	0.67	94.1
Soybean	5.7	0.06	81.5	0.41	93
Sunflower	6.4	0.05	105.3	0.68	93.9
Vetch	6.5	0.04	112.7	0.38	94.2

## Root Elongation

### Penetration of strong layers by roots

The intra‐specific discrimination between roots can be obtained by measuring the ability of a root to penetrate a hard layer (Clark *et al.,* 2002; Chimungu *et al.,* 2015). Hard layer penetration is commonly tested using wax layers which can be prepared to different strengths by melting together different amounts of soft and hard wax. There is some evidence that the ability to penetrate a hard layer is related to improved performance of cultivars in water limited conditions (Botwright Acuña *et al.,* 2012). Apart from providing a greater discrimination between cultivars than other screens, the hard‐wax‐layer method provides an intuitive experimental model of hard layers in the soil, frequently referred to as ‘pans’. So‐called ‘pans’ can either be natural features which limit water uptake from depth (Shanahan *et al.,* 2015) or they can develop over time in cultivated systems and are referred to as ‘plough‐pans’. Plough‐pans sometimes form when tractor tyres run in the bottom of the plough furrow and compact soil at the ploughing depth (between 20 and 30 cm). However, a more common cause is the inevitable use of blunt plough shares which force some soil downward. Although there is little supporting evidence, it is often assumed that roots with a good ability to penetrate hard layers in the laboratory will be better at penetrating through plough pans in the field.

### Soil structure and root elongation

It is probable that the laborious nature of the measurements has led to relatively few reports of root elongation in relation to soil structure and soil depth; however, those measurements which have been published (White & Kirkegaard, 2010) show that at depth (>90 cm) all roots were found in pre‐existing pores or cracks. Similar conclusions were drawn from data recently obtained at Rothamsted. Another important conclusion to be drawn from the data published by White & Kirkegaard (2010) is that it is only in the shallower soil layers that roots are capable of elongating by deforming the soil with the processes modelled by Kirby & Bengough (2012). The data of White & Kirkegaard (2010) are entirely consistent with both the effect of increasing penetrometer resistance with depth (Fig. 2) and the published data showing poor root elongation at high values of penetrometer resistance (Table 1). A particularly noteworthy finding from White & Kirkegaard (2010) is that at a depth of 1 m only 5% of pores contain roots indicating that either roots are poor at locating pores or that there is no continuity of pores between the lower and upper layers. Wang *et al.* (1986) found that if roots of soybean (*Glycine max*) did not meet macropores before a depth of 30 to 45 cm then the root tips died. However, roots which extend into burrows followed them to their end. Ehlers *et al.* (1983) found that although soil strength was greater in the surface of no‐till soils, there was no reduction in root length density because of roots growing in burrows.

In a comparison of 17 different wheat lines at two different field sites, Wasson *et al.* (2014) found little effect of genotype in determining rooting depth, the amount of shallow roots or the amount of deeper roots. However the ratio of roots deeper than 130 cm to total root length was significantly affected by genotype. The field sites (i.e. soil type) had the greatest effect on the distribution of roots with depth, with one of the sites encouraging a much greater root length density at depths shallower than approximately 1 m in all of the wheat lines.

A comparison between oats grown on tilled and untilled soil is described by Ehlers *et al.* (1980). The root length distributions with depth were very similar, except that the tilled treatment allowed a greater root length in the shallower layers and early shoot growth was more vigorous. Later in the season there was greater water uptake from deeper layers in the untilled plots. There was very little difference in the final yield, although the temporal growth patterns were different because of different root length distributions with depth. Thus soil management offers a way to regulate the water supply over a season, although in Germany where this study was made, this is less important than it would be in a semi‐arid region. Regulation of water use during the season can also be achieved by breeding wheat with a less conductive xylem (Richards & Passioura, 1989), which emphasizes the opportunity for complex interactions between the crop and environment.

### Deep roots in laboratory studies

**Figure 5:**
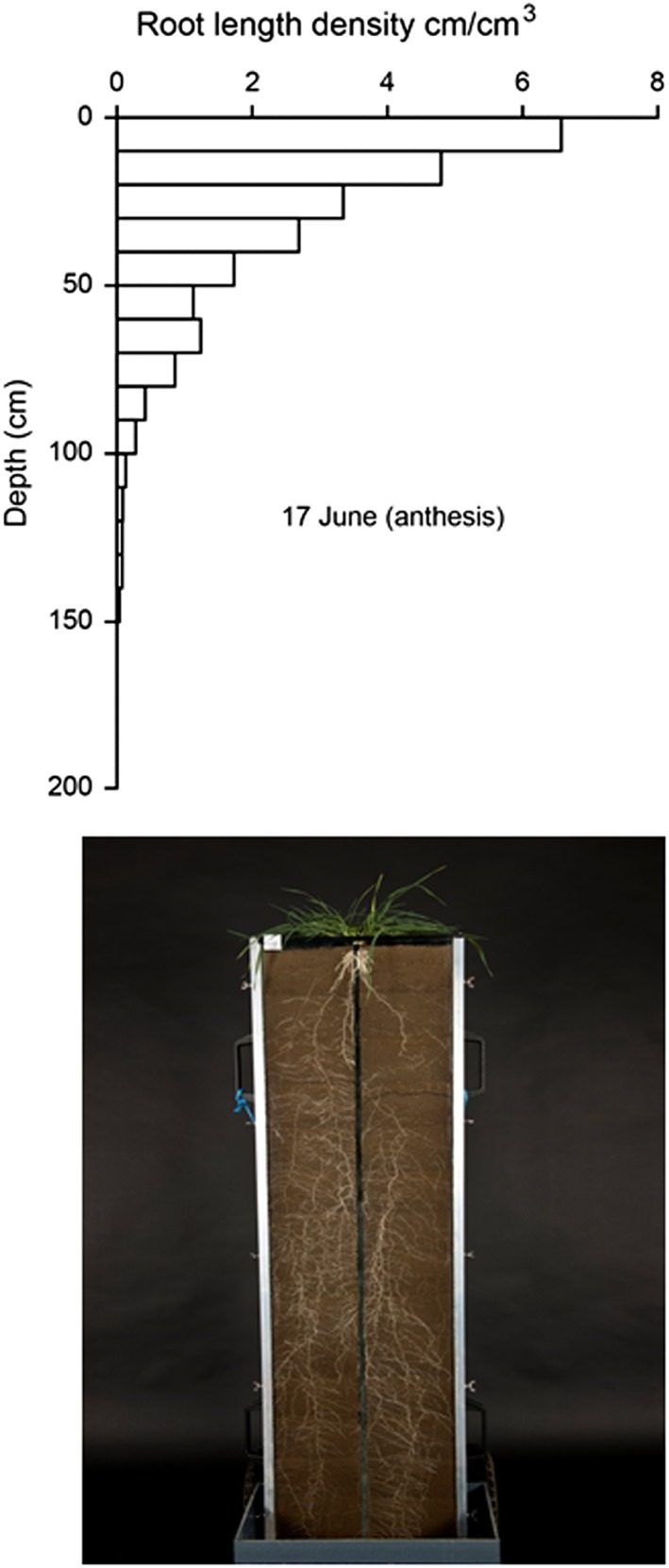
Comparison of wheat root distributions with depth from rhizotons and from data collected from a field experiment. The photograph is from a rhizotron experiment at Rothamsted while the field data was published by Gregory *et al.* (1978). The rhizotron image shows very little gradient in root mass with depth, and similar data have been published by Manschadi *et al.* (2008). In the field, root length density decreases rapidly with depth; this is a typical result. The rhizotron was 1.4 m in height.

Many accounts of root elongation in the laboratory show considerable root growth at depth (e.g. Manschadi *et al.,* 2008). However, such data are usually obtained from a laboratory rhizotron arrangement, where the soil is packed to a given density and is probably warmer than soil at depth in the field. Although, these often replicate the depth of soil in the field (e.g. Jin *et al.,* 2015b) for reasons of practicality their dimensions are limited and can be in the order of 10 cm thick. In a long and narrow column the weight of the soil is supported by the friction between the soil and the walls and it is not transmitted down to the base of the rhizotron. In agriculture the best example of this is to be found in grain silos where in very tall silos the weight of the grain is actually supported by the walls and not the concrete base (Marchant & Westgate, 1982). The same principle applies to tall rhizotrons as well as long narrow tubes packed with soil. In many respects rhizotrons have produced important data, for example the angular spread of wheat roots (Manschadi *et al.,* 2008), but it is likely that rooting depth inferred from these experimental systems does not reflect the situation in the field with respect to soil strength at depth. Comparisons of root length density for wheat measured in the field by Gregory *et al.* (1978) and our images of root systems from rhizotron studies show clear evidence of an inconsistency (Fig. 5).

### Very deep roots in field studies

Although Jackson *et al.* (1999) show that deep rooting to depths of 10 s of metres is common in the natural environment for some species, it is almost certainly the case that these roots exploit structural pores connected to great depths. In their review, Canadell *et al.* (1996) found that some species growing in dry conditions had particularly deep roots. They noted that a commonly held view was that very deep roots could only be found in sandy soils, a view they contested in their paper pointing out that deep roots had also been reported to penetrate compacted clay. Our analysis suggests that in clay soils very deep roots are unlikely to be the results of soil deformation. However, shrinkage of clay soils by forces developed during desiccation because of root water uptake may create structure that can be exploited by roots, especially in perennial systems. Canadell *et al.* (1996) comment that penetration of roots into bedrock, which would be the case for roots detected in deep caves, was probably by the exploitation of fissures and cracks. With respect to sand, Whalley *et al.* (1999) found that roots of carrot seedlings were not affected by mechanical impedance in sand culture systems. This was almost certainly because the fine carrot roots were small enough to elongate through the sand's pores with ease. This is likely to be the mechanism which allows very deep rooting in sands, where Canadell *et al.* (1996) report roots to a depth of 53 m. Contrary to the commonly held view, provided there has not been excessive drying, clay soils offer a lower impedance to root elongation than sands (Gregory *et al.,* 2007). Indeed Shanahan *et al.* (2015) showed that water uptake at depth can be greater in clay soils compared to sandy soils.

It should be noted that in this article our primary interest is in cultivated agricultural soils. The interaction between plant roots and soil in natural systems evolves over much longer time scales and is more complex than in agriculture. Some of these interactions in natural ecosystems are outlined by Verboom & Pate (2013), who suggest that rooting depths may depend on processes that occur over geological time scales, such as erosion, weathering of minerals as well as the effect of biological system. In this case deep rooting is not due simply to soil deformation or pore location, but is the result of complex interactions that occur over long time scales.

### Location of pores by roots

We are making the case that that deep roots can only be found when they are able to exploit existing pore networks. These could be old root channels, earthworm channels or structural areas of weaker soil that can occur in soils with high clay content. Old root channels might be legacy features following perennial plant/crop cover. While earthworms are widely believed to be an important source of biopores, interestingly, they are only able to exert relatively modest axial or radial pressures (McKenzie & Dexter, 1988a, 1988b; Stovold *et al.,* 2003) and their primary mode of burrowing is not soil deformation, but soil ingestion and transport. If deep roots have to exploit these pore structures, then a key root trait to confer deep rooting may not be the ability to deform strong layers, but to locate existing pore networks. This trait has been described by Dexter (1986) and called trematotropism. Dexter (1986) noted that there was little evidence for roots preferentially locating pores in well‐aerated soil, although there was more limited evidence in poorly aerated soil. Stirzaker *et al.* (1996) found that barley grew better in soil with a network of narrow biopores created by lucerne or ryegrass compared with larger artificially constructed pores. Intriguingly, they observed that roots responded positively when biopores were filled with peat. A particularly interesting hypothesis that worm casts deposited in burrows may stimulate plant roots to elongate preferentially to those burrows was explored by Hirth *et al.* (1997); however, their data did not support the hypothesis. Their study was stimulated by a report from Springett & Syers (1979) that roots of ryegrass seedlings that were only eight days old elongated preferentially to earthworm casts.

In an interesting field study, McKenzie *et al.* (2009) compared the ability of different barley lines to find and elongate through pores at different densities (pores m^−2^). The pores were created by burying a two‐dimensional geotextile at 20 cm, with the different pore‐density treatments. Although no genotypic differences were found, this approach would seem to provide a method to assess genotypes. Either McKenzie *et al.* (2009) were unlucky with their choice of genotypes or the process of a root finding a pore can only be treated as a three‐dimensional problem. Indeed, the observation by Stirzaker *et al.* (1996) that roots are more effective at exploiting old root channels than artificially created pores suggests that relationship between the geometry of the pore network and the architecture of the root system is important. The improving ability to make CT X‐ray images of larger soil cores (Tracy *et al.,* 2015) will become increasingly important.

The basis for the location of soil pores by roots seems to be a relatively unexplored area and given the increases in soil strength with depth (Fig. 3) it would appear to have the potential to be a productive line of enquiry. It seems likely that the probability of roots encountering a pore depends on the degree of branching in a root system as well as on pore density and distribution. Root branching can be related to genetics, but also influenced by the physical environment. Chapman *et al.* (2011) found that the number of secondary roots in Arabidopsis increased with the hydraulic conductance of the soil. Atkinson *et al.* (2015) also report a strong environmental effect on root branching, and they also identify the interaction between root branching, other root traits and the environment as a major challenge to be addressed.

### Is the ability of roots to penetrate hard layers important?

If we accept the thesis that deep root penetration is facilitated by exploiting existing pore networks, then the question arises of whether an ability to penetrate a hard layer is useful. Actually, we maintain that it is useful. Roots which deform soil are likely to have better root–soil contact and improved ability to extract water and nutrients from the soil in the shallower layers. At depth, roots in pores are less well connected hydraulically to soil, although White & Kirkegaard (2010) show that roots elongating in large pores can be connected to the soil by root hairs. When more than one root occupies soil pores, so called ‘root clumping’, roots become distributed in clusters which is less effective at draining soil than uniformly distributed roots (Tradieu *et al.* 1992). The ability of clumped roots to drain soil depends on the spacing of the biopores, because of old roots and earthworms (Passioura, 1991). Unfortunately, although biopores seem to be the most common structure to enable deep rooting, Passioura (1991) showed that their spatial geometry was the least effective for allowing soil to be dried by roots.

## Concluding Remarks

While the tendency for deeper roots to be found in pores is well reported (e.g. Lynch & Wojciechowski, 2015), we provide an explanation for why this is inevitable. The confinement of deeper roots to existing pore networks is almost certainly related to the increased soil penetrometer resistance that occurs with depth even in soils that have not been damaged by compaction. We have demonstrated that this effect can occur in relatively shallow soil (50 cm), but it is exacerbated by compaction. The ability of roots to penetrate hard layers is unlikely to be correlated with very deep rooting, although it is still a useful trait and likely to be associated with better exploration of surface layers and water or nutrient uptake. Penetration by roots into deeper layers is likely to depend on how well roots are able to find existing pore networks and we suggest that this question needs greater attention. The greater depth of roots that can be found in natural systems compared to cultivated soils illustrates the importance of soil structure in facilitating deep rooting. While large differences in rooting depth between different cultivars of the same species are reported, differences in soil type and management are likely to be more important factors than genotype. When comparisons of rooting depth between different genotypes have been made in the same soil, the reported differences in rooting depth have been small. Presently we do not know if the ability of roots to locate pores is simply stochastic or whether there is an underlying biological mechanism. It is also unclear how differences in root architecture and soil structure interact to determine how effectively roots locate pore networks. However, once the mechanism is understood it would aid breeding for deep rooting and improved water and N uptake.
